# Supplementary material for: Considering Hemispheric Specialization in Emotional Face Processing: An Eye Tracking Study in Left- and Right-Lateralised Semantic Dementia
Source: Brain Sci. 2021 Sep 10;11(9):1195. doi: 10.3390/brainsci11091195 (PMC8472320; doi:10.3390/brainsci11091195)
Supplement: Supplementary file 1 [file brainsci-11-01195-s001.zip › brainsci-1322579-supplementary.pdf]

Supplementary Materials

Duration of fixations

Duration of fixations was also examined, in order to explore whether differences in fixation number between groups was related to differences in time spent viewing these regions.[1] Duration of fixations to the whole face did not differ between groups ($F(2,32) = 1.018$, $p = .373$).

Data for duration of fixations to the eyes and mouth are shown in Figure 4. Duration of fixations to the eyes showed a similar pattern of performance as that observed for number of fixations; however, the main effect of group was not significant ($F(2,35) = 0.705$, $p = .501$, $\eta_p^2 = .039$). A main effect of emotion was evident ($F(2,70) = 25.721$, $p < .001$, $\eta_p^2 = .424$), with participants spending less time fixating on the eyes in the happy condition, than both the fear and neutral (both *p* values < .001) conditions and no difference between fear and neutral conditions ($p = .894$). Again, the interaction between group and emotion was not significant for duration of fixations to the eyes ($F(4,70) = 0.262$, $p = .901$, $\eta_p^2 = 0.14$).

[1]As for number of fixations, outliers more than 2 standard deviations away from the group mean were excluded from the relevant analyses. This included three outliers (one from each group) in the whole face analysis and two outliers (1 control, 1 left-SD) in mouth analysis.

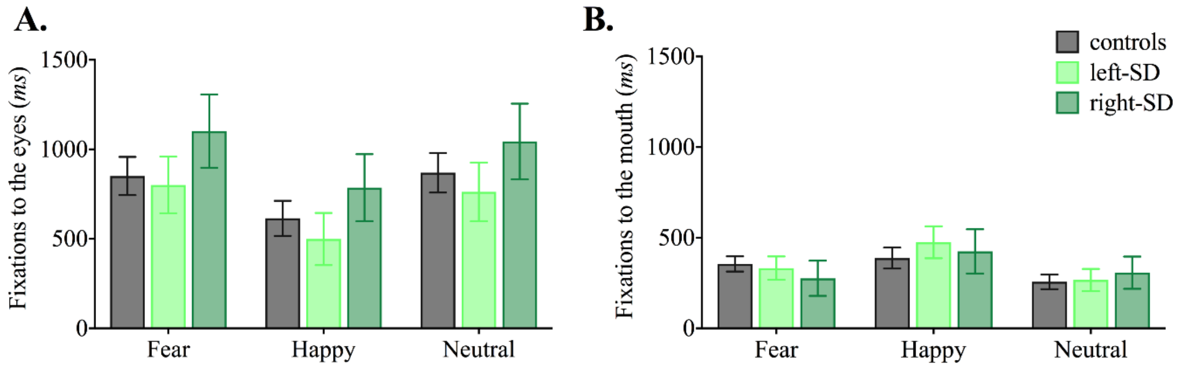

Supplementary Figure S1. Duration of fixations, in milliseconds (*ms*), to the (A) eyes and (B) mouth shown by controls (grey), left-lateralised semantic dementia (left-SD, light green) and right-lateralised semantic dementia (right-SD, dark green) when viewing faces expressing fear, happy or neutral.

For duration of fixations to the mouth, no difference between groups was evident ($F(2,33) = 0.085$, $p = .919$, $\eta_p^2 = .005$). The main effect of emotion was significant ($F(2,66) = 10.381$, $p < .001$, $\eta_p^2 = .239$), where a longer duration of fixations was observed in the happy condition, compared to the fear ($p = .017$) and neutral ($p = .002$) conditions, with no difference between fear and neutral ($p = .318$). No interaction between group and emotion was evident for duration of fixations to the mouth ($F(4,66) = 0.875$, $p = .484$, $\eta_p^2 = .050$).

Voxel-based morphometry analysis by emotion

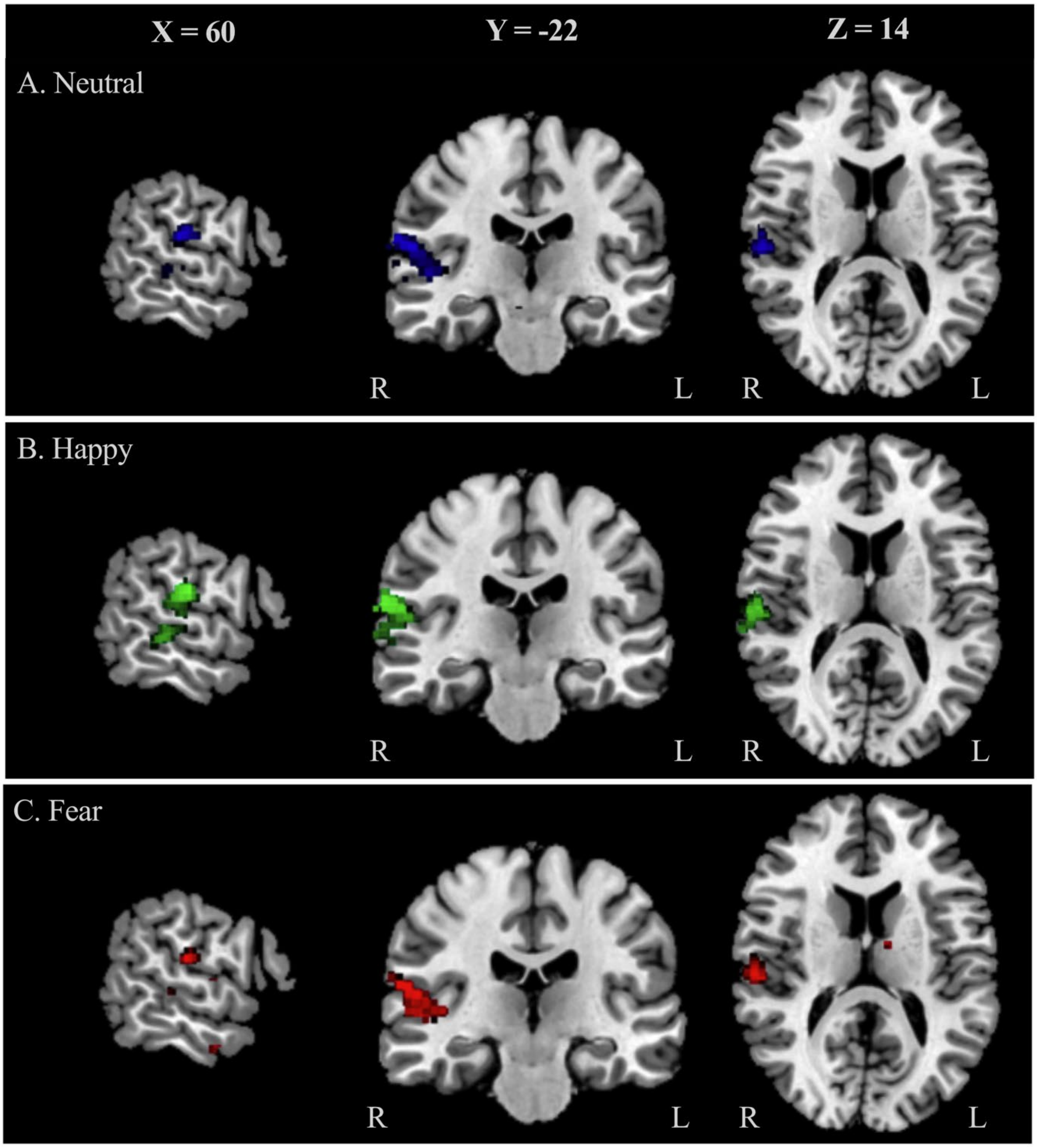

Supplementary Figure S2. *V*oxel-based morphometry analyses showing brain regions where reduced grey matter intensity correlated with more fixations to the eyes in the neutral (A; blue), happy (B; green) and fear (C; red) conditions in left-lateralised semantic dementia, right-lateralised semantic dementia and controls combined. Coloured voxels show regions that were significant in a voxel-wise analysis at $p < .005$, uncorrected, with a cluster extent threshold of 150 voxels. R = right; L = left. MNI coordinates: x = 60; y = -22, z = -14.

Supplementary Table S1. Voxel-based morphometry results showing significant negative correlation between grey matter intensity and fixations to the eyes each for each emotion in all semantic dementia patients and controls combined.

Regions	Hemisphere	MNI coordinates			Number of voxels
		x	y	z	
A. Neutral					
Parietal operculum cortex, planum temporale, extending into the central opercular cortex, supramarginal gyrus, Heschl's gyrus (H1 and H2)	right	60	-24	16	297
B. Happy					
Supramarginal gyrus, extending into the parietal operculum cortex, planum temporale, central opercular cortex, post central gyrus, superior temporal gyrus, middle temporal gyrus	right	68	-22	18	503
C. Fear					
Insula extending into Heschl's gyrus, planum polare, superior temporal gyrus (posterior), planum temporale, parietal operculum cortex, supramarginal gyrus (anterior)	right	42	-8	2	413

Note: Results are voxel-wise and reported at $p < .005$ uncorrected for multiple comparisons, with a cluster threshold of 150 voxels.
